# Enhancing Bioactive Saponin Content of *Raphanus sativus* Extract by Thermal Processing at Various Conditions

**DOI:** 10.3390/molecules27238125

**Published:** 2022-11-22

**Authors:** Min Yang, Chih-Yao Hou, Hsien-Yi Hsu, Sulfath Hakkim Hazeena, Shella Permatasari Santoso, Cheng-Chia Yu, Chao-Kai Chang, Mohsen Gavahian, Chang-Wei Hsieh

**Affiliations:** 1 Department of Food Science and Biotechnology, National Chung Hsing University, 145 Xingda Rd., South Dist., Taichung 40227, Taiwan; 2 Department of Seafood Science, National Kaohsiung University of Science and Technology, 142, Haizhuan Rd., Nanzi Dist., Kaohsiung 81157, Taiwan; 3 Department of Materials Science and Engineering, School of Energy and Environment, City University of Hong Kong, Hong Kong 999077, China; 4 Shenzhen Research Institute, City University of Hong Kong, Shenzhen 518057, China; 5 Department of Chemical Engineering, Widya Mandala Surabaya Catholic University, Surabaya 60114, Indonesia; 6 Department of Chemical Engineering, National Taiwan University of Science and Technology, Daan Dist., Taipei 10607, Taiwan; 7 School of Dentistry, Chung Shan Medical University, No.110, Sec.1, Jianguo N. Rd., Taichung 40201, Taiwan; 8 Institute of Oral Sciences, Chung Shan Medical University, No.110, Sec.1, Jianguo N. Rd., Taichung 40201, Taiwan; 9 Department of Dentistry, Chung Shan Medical University Hospital, No.110, Sec.1, Jianguo N. Rd., Taichung 40201, Taiwan; 10 Department of Food Science, National Pingtung University of Science and Technology, Pingtung 91201, Taiwan; 11 Department of Medical Research, China Medical University Hospital, Taichung 404333, Taiwan

**Keywords:** bioactive compounds, extract, thermal treatment, radish, *Raphanus sativus*, saponins

## Abstract

Pickled radish (*Raphanus sativus*) is a traditional Asian ingredient, but the traditional method takes decades to make this product. To optimize such a process, this study compared the saponin content of pickled radishes with different thermal processing and traditional processes (production time of 7 days, 10 years, and 20 years) and evaluated the effects of different thermal processes on the formation of radish saponin through kinetics study and mass spectrometry. The results showed that increasing the pickling time enhanced the formation of saponin in commercial pickled radishes (25 °C, 7 days, 6.50 ± 1.46 mg g^−1^; 3650 days, 23.11 ± 1.22 mg g^−1^), but these increases were lower than those induced by thermal processing (70 °C 30 days 24.24 ± 1.01 mg g^−1^). However, it was found that the pickling time of more than 10 years and the processing temperature of more than 80 °C reduce the saponin content. Liquid chromatography–mass spectrometry (LC-MS) analysis showed that the major saponin in untreated radish was Tupistroside G, whereas treated samples contained Asparagoside A and Timosaponin A1. Moreover, this study elucidated the chemical structure of saponins in TPR. The findings indicated that thermal treatment could induce functional saponin conversion in plants, and such a mechanism can also be used to improve the health efficacy of plant-based crops.

## 1. Introduction

Radish (*Raphanus sativus*) belongs to the plant family Brassicaceae and is an important vegetable with high nutritional and bioactive properties. In East Asia, radish roots are pickled with salt to produce a tasty product that is believed to be beneficial in curing liver and respiratory illnesses. Related research has pointed out that *R. sativus* contains alkaloids, nitrogen compounds, coumarins, gibberellins, glucosinolates, organic acids, phenolic compounds, polysaccharides, proteoglycan, and sulfur compounds [1]. Among these, the bioactive compounds are probably responsible for their therapeutic effects. For example, *R. sativus* was found to have bioactive compounds, such as vitamins C, lignin, phenols, flavonoids, and isothiocyanates [2]. The sulforaphene in *R. sativus* could inhibit cell proliferation of human cancer cells similar to in Chinese herbal medicines [3]. Some studies also showed that *R. sativus* has bioactivity, such as anti-microbial, anti-oxidant [2,4], anti-inflammatory, and anti-diabetic properties [2,5]. The above are among the reasons for the use of *R. sativus* extracts in traditional and modern medicines [6,7]. Similar observations were made for other plant materials. For example, the olive fruit was mostly used for oil extraction, but recent studies found that extracts from olive leaf have antioxidant, antimicrobial, and anticancer activities. In a recent study, olive leaf extract obtained by water extraction was used to reduce acrylamide, to increase phenols, and to assist table olives fermented [8,9,10]. These highlight that phytochemicals have great potential in the development of healthy products.

Saponins are widely present in the plant kingdom and can function as natural bio-surfactants due to their amphiphilic structure that contains both lipophilic aglycones and hydrophilic sugar moieties. Moreover, saponins are applied in food, cosmetics, chemicals, and pharmaceuticals as a potential drug delivery system [11]. Previous studies demonstrated that varieties of *R. sativus* are rich sources of saponins. For instance, the phytochemical analysis revealed the presence of saponins in *R. sativus* extracts from methanol, ethanol, ethyl acetate, and petroleum ether [12]. The hot water extract of *R. sativus* contained saponins that could ameliorate atherogenic lipid profiles in hyper-cholesterolemic rats and lower the cardiovascular disease risk factors [13]. Other results demonstrated that methanolic extracts of *R. sativus* contain saponin that acts as an antibacterial substance, especially to prevent *Streptococcus sanguis* [14]. So far, in the Brassicaceae family, the saponin structure was analyzed only in *Barbarea vulgaris* and *Lepidium meyenii* [15]. In *Barbarea vulgaris*, the structure was found as 3-O-cellobiosyl-hederagenin, 3-O-cellobiosyl-gypsogenin, and 3-O-cellobiosyl-4-epihederagenin, which were isolated and characterized for insect resistance and the development of natural insecticides in food production [16]. Among a total of 25 chemical compounds extracted from the root of *Lepidium meyenii*, the structure of saponins (tanshinone I, panaxytriol, oleanoic acid, and rotundifolioside) was identified [17]. In addition to the positive biological effects of saponins, such as hypocholesterolemia, anti-inflammatory, antitumor, immunomodulatory, antibacterial, antiviral, antifungal, and antiparasitic activities [18], they have antinutrients activities due to their hemolytic activity, inhibitory activity of digestive enzymes, and their effect on the permeability of the small intestinal mucosal cells [19]. Therefore, some studies explored the approaches to remove saponin. For example, it has been recommended to remove the surface saponins of quinoa (*Chenopodium quinoa*) by a grinding process [20]. However, the literature mainly focused on the beneficial effects of saponins as affected by processing; for example, by increasing absorbability and the antibacterial ability of saponins [21,22]. At present, most of the ways to obtain saponins in plants need to use organic solvents, such as extracting spirostanol saponins from *rhizomes* with petroleum ether and ethanol. [23,24]. In view of the development of diet therapy, more and more studies have focused on the influence of traditional food processing on the structure of naturally present food components.

Thermal treatment is a common processing method that has been used for developing products such as black olives. This product is made by temperature control and long-term immersion, which can improve the function of olives. However, the traditional process of black olives also takes several months and can easily lead to oxidation and deterioration in the olives [8,9,10]. Therefore, the feasibility of shorten the process time of plant materials and their ability to improve their functionality is a rather interesting research topic. Related studies have also confirmed that thermal treatment can reduce the content of saponins, bitter substances, and antinutrients [25]. This could be induced by the thermal degradation of these compounds and thermal-induced structural changes [26]. On the other hand, such a process could increase the bioactive contents and modify the composition of foods and traditional Chinese medicines [21,24,27]. For instance, in *Ginkgo biloba* seeds treated by thermal processing, 4′-O-methylpyridoxine analogs content increased, which demonstrated important biological activities, such as immunomodulatory, antitumor, and anti-oxidative activities [28,29]. Moreover, high-temperature and high-pressure thermal processing increased the bioactive components of ginsenosides that were conversed from ginseng. Among the ginsenosides (Rb1, Rb2, Rc, Rd, and Re), the deglycosylation of Rd improved the anticancer activity of the root of ginseng [30]. In the red ginseng processing, the high steaming temperature (120 °C) can promote the transformation of less polar ginsenosides, such as 20R-Rs3 and Rs4 [31], that might develop new types of antibacterial substances and skin care cosmetics for acne prevention and therapy [21]. Thermal treatment can change the structure of saponins through deglycosylation or the transformation of less polar ginsenosides, and bioactivity can be enhanced. Moreover, the negative biological effects of saponins can also be reported, which could be detoxified by thermal treatment [22,32].

Similar observations were made for other plant materials. Though it has been reported that thermal processing could increase the concentration of phenols, flavonoids, and antioxidant properties in radishes [33], there is no research discussing the composition changes of saponins in *R. sativus* after thermal treatments at various temperatures. This study aimed to analyze the saponin content of thermally processed radish (TPR) and traditionally commercial pickled radish and to analyze the effect of thermal processing on the structural changes of radish saponin by kinetics and mass spectrometry. Additionally, it is expected to provide a basis for agricultural product processors to optimize traditional processes of radish.

## 2. Results and Discussion

### 2.1. Effects of Different Processing Methods on Saponin Formation in Radish

Table 1 shows the results of saponin content of heat-treated radish in comparison with commercially available pickled radish. According to the results, saponin content increased with the increase of storage time from 7 days (CPR A 6.5 mg g*^−^*^1^) to 3650 days, (CPR B 23.11 mg g*^−^*^1^). However, the saponin content of radish prepared at 25 °C decreased to 18.37 mg g*^−^*^1^ (CPR C) during a storage time of 7300 days. Previous studies have pointed out that excessive pickling and dehydration may lead to the loss of saponin in vegetables [11]. Similar observations were made in the present analysis of commercially available pickled radishes when the pickling time is too long. On the other hand, the TPR group showed that the saponin content in the radish was similar to that of the CPR B group when the radish was treated at 70 °C for 30 days (24.24 mg/g). Relevant studies have pointed out that the content of ginsenosides in the ginseng after 130 °C and 4 h in the autoclave will increase the content of saponins released from the tissue due to the destruction of the cell structure [21].

However, when the temperature used is 80 °C, it will decrease (although the saponin content is similar to the CPR B group) to 23.33 ± 2.32 mg g*^−^*^1^. Previous reports pointed out that thermal treatment can reduce the content of saponins, some bitter substances, and antinutrients [25,26]. This result is not entirely due to the loss of saponin, and part of the reason may be due to the transformation of the structure of saponin. Further research also pointed out that after thermal treatment of ginsenoside, the degradation or repolymerization of saponin can help improve the bioavailability of saponin, thereby significantly improving the antibacterial and anti-inflammatory properties of saponin [21]. Therefore, we further analyzed the saponin structure of the TPR group to explore the structural changes of saponin after the thermal treatment of radish, and the benefits of thermal treatment in terms of improving radish functional properties.

### 2.2. Saponin Content as Affected by Different Thermal Processing

**Figure 1:**
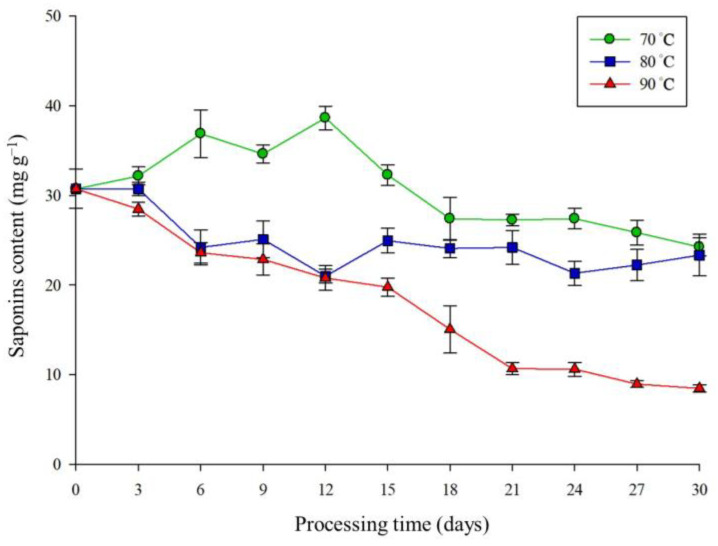
Changes in the saponin content of radish during thermal processing. Thermally processed radish (TPR) was treated at 70 °C, 80 °C, or 90 °C from 0 to 30 days. The error bars represent the standard deviation (*n* = 3).

To study the effect of thermal processing on the saponin content of TPR, we heated the radish at various temperatures, including 70 °C, 80 °C, and 90 °C, and the samples were collected at 3 days intervals for saponin analysis. The results showed the saponin concentrations of radish that were processed at 70 °C for 0, 12, and 30 days were 30.71 mg g*^−^*^1^, 38.63 mg g*^−^*^1^, and 24.24 mg g*^−^*^1^, respectively (Figure 1). It was observed that the levels of saponins were reduced from 23.33 mg g*^−^*^1^ to 8.47 mg g*^−^*^1^ for 30 days at 80 °C and 90 °C, respectively. These observations showed that thermal processing would have significant effects on the saponin content in the radish. Compared with day 0, the thermal processing at 70 °C and 80 °C for 30 days retained 80% of saponin content in the radish. However, the saponin content of TPR continuously decreased while the processing was conducted at 90 °C.

Previous studies pointed out that saponin is a heat-sensitive substance, and saponin may be cracked or resynthesized under the action of heat [25,26]. As shown in Figure 1, when radish was treated at 70 °C, saponin could be more easily released from the cell tissue in the early stage of storage (the first 15 days) due to proper tissue destruction, and the content of saponin might decrease due to thermal damage in the subsequent period. The 80 °C and 90 °C treatment groups were destroyed due to excessive temperature, which shows that the temperature has a more significant effect on the saponin in radish. Previous studies also showed that thermal processing may influence the saponins’ content [21,26]. For instance, the thermal sensitive saponins in bitter melon, 3β,7β, 25-trihydroxycucurbita-5,23(E)-dien-19-al, and momordicine I could significantly decrease after 5 mins of thermal processing at 100 °C [25]. Other saponins, such as momordicoside L, momordicoside K, momordicoside I, and momordicoside F1, remained stable during treatment at 60 °C for 20 mins [25]. In addition, the thermal treatment would partially eliminate the bitter taste of saponins in melon [25]. Thermal processing at 100 °C on navy beans also had greater effects on saponins B degradation, showing that the excess thermal treatment could have negative effects on saponin content [26]. The other results showed that thermal processing at 121 °C on colorful bean (Phaseolus vulgaris L) could increase the soyasaponin Ba and I concentration and decrease soyasaponins αg and βg, supporting the finding that thermal processing can retain the saponin content [34]. Moreover, the previous study also showed that soyasaponin Ba has remarkable bioactivity [35]. In the red ginseng processing, the heat process could promote the transformation of polar ginsenosides to less polar, which is easier to be absorbed by the human body, and, thus, can be more bioavailable [21]. These previous studies indirectly pointed out that 70–90 °C treatment of radish may not only affect the content of saponin, but also may change the structure of saponin.

### 2.3. Kinetics of Saponins’ Formation at Different Thermal Treatment Temperatures

Table 2 shows that the K value of the 70 °C treatment group is positive in both zero-order (0.1820) and first-order (0.0050) kinetics, and is greater than 80 °C and 90 °C, indicating that the 70 °C treatment is the most favorable temperature for the formation of radish saponin. Table 2 shows that the analysis of the zero-order (R^2^ = 0.7181), first-order (R^2^ = 0.7507), and second-order (R^2^ = 0.7821) kinetics of the 70 °C treatment groups showed a high correlation. It has been demonstrated that the zero-order reaction means that the product contained only accounts for a small part of the reactant, and because the reactant content is much more than the product, a stable concentration can be maintained during the reaction process. The secondary reaction represents that the product also plays the role of a catalyst, which can increase the reaction rate while generating. For example, when milk is heated, lysine will react with lactose to produce a chemical change in the Mena reaction. During this process, lysine can be regenerated, and produce a catalytic effect, which further produces higher-order Mena reactions [36,37].

This shows that when the radish is treated at 70°C, the formation of saponin can be stably produced, the reaction rate is faster, and the temperature or other compounds may play the role of catalyst. This also explains the increase in saponin before day 12 in the 70 °C treatment group in Figure 1. The correlation analysis in the 80 °C treatment group is more inclined to zero-order kinetics (R^2^ = 0.7126), which may be because the reaction of saponin under thermal destruction is greater than the synthesis of new saponin, so the concentration of the product is always lower than the initial saponin concentration (Figure 1.). Table 2 also shows that the 90 °C treatment has a high correlation with the second-order kinetics, which means that the high degree of catalyst in this module interferes with the formation of saponin. Figure 1 shows that the saponin content decreased rapidly in the early stage of thermal treatment. Therefore, the kinetic analysis results in Table 1 show that the treatment at 90 °C is the key factor affecting the reduction of saponin content.

### 2.4. Identification of the Saponin Composition in TPR

**Figure 2:**
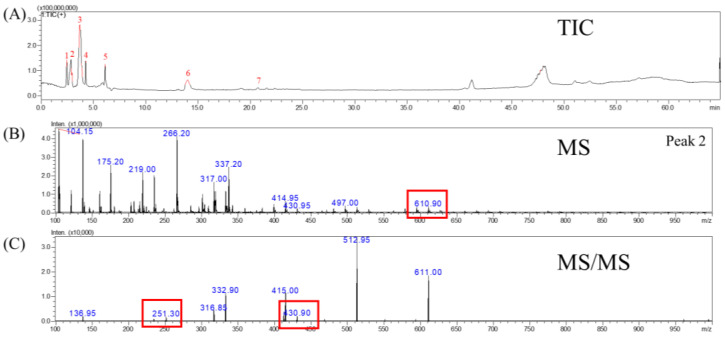
The mass spectrometry of (**A**) total ion chromatogram (TIC), (**B**) MS spectra, and (**C**) MS/MS spectra of the molecule under positive ion mode at *m*/*z* 610.90 from the untreated radish.

**Figure 3:**
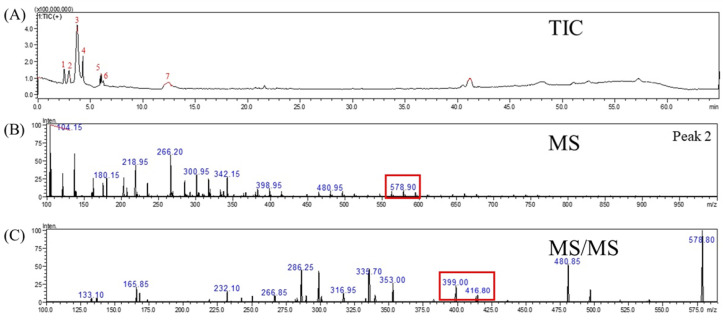
The mass spectrometry of (**A**) total ion chromatogram (TIC), (**B**) MS spectra, and (**C**) MS/MS spectra of the molecule under positive ion mode at m/z 578.90 from the thermally treated radish at 70 °C for 12 days.

**Figure 4:**
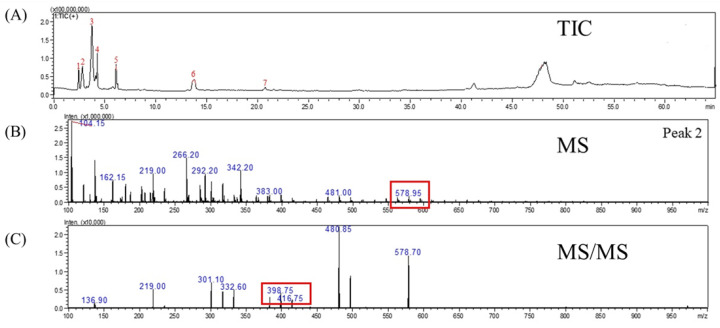
The mass spectrometry of (**A**) total ion chromatogram (TIC), (**B**) MS spectra, and (**C**) MS/MS spectra of the molecule under positive ion mode at *m*/*z* 578.95 from the thermally treated radish at 70 °C for 30 days.

**Figure 5:**
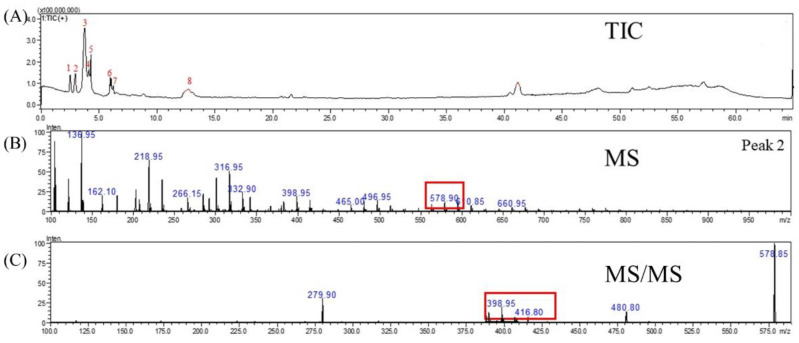
The mass spectrometry of (**A**) total ion chromatogram (TIC), (**B**) MS spectra, and (**C**) MS/MS spectra of the molecule under positive ion mode at *m*/*z* 578.90 from the thermally treated radish at 80 °C for 30 days.

Tupistroside G, Asparagoside A, and Timosaponin A1 were identified as the major saponins in TPR using HPLC-MS/MS. Figure 2A, Figure 3A, Figure 4A and Figure 5A showed all mass spectral peaks by total ion chromatogram (TIC) in the groups of the untreated radish, TPR (at 70 °C for 12 days), TPR (at 70 °C for 30 days), and TPR (at 80 °C for 30 days), respectively. Individual saponins of TPR, detected from peak 2 in Figure 2A using LC-MS, are shown in Figure 2B. In the positive ion mode, the molecular ion peak with *m*/*z* 610.90 ([M + H]^+^) could be distinguished from the saponins. Based on the HPLC-MS/MS data, the fragment ion at *m*/*z* 430.90 and 251.30 could be Tupistroside G in Figure 2C. Figure 3B showed that TPR (at 70 °C for 12 days) in the positive ion mode and the molecular ion peak with *m*/*z* 578.90 ([M + H] ^+^) could be distinguished from the other saponins by the database. With the HPLC-MS/MS data in Figure 3C, the fragment ion at *m*/*z* 416.80 and 399.00 represented Asparagoside A and Timosaponin A1, respectively. A similar result of TPR treatment, at 70 °C for 30 days, was also shown in Figure 4B, which obtained the structure of saponins in the molecular ion peak with *m*/*z* 578.95 ([M + H] ^+^) by HPLC-MS and produced the fragment ion at *m*/*z* 498.75 and 416.75, which represent Asparagoside A and Timosaponin A1 by HPLC-MS/MS in Figure 4C. In addition, TPR treated at 80 °C for 30 days could obtain the saponin structure in the molecular ion peak with *m*/*z* 578.90 ([M + H] ^+^) in Figure 5B and the fragment ion at *m*/*z* 398.75 and 416.75, which represent Asparagoside A and Timosaponin A1 in Figure 5C. Saponin is a heat-sensitive substance, and the above results showed that saponin experienced significant structural changes after thermal treatment. These structural changes may reduce the polarity of saponin due to the loss of a portion of the hydroxy group [31], which further affects the performance of the functional components of saponin.

**Figure 6:**
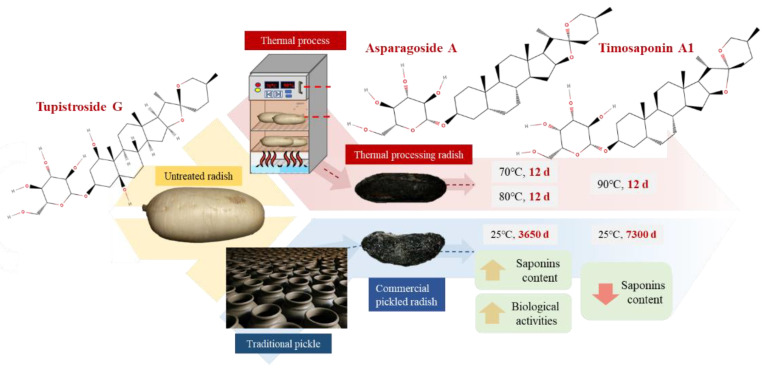
A summary of the methods and main findings of this study. The molecular formula was drawn by the author with MolView software.

According to the literature, Tupistroside G could be extract) would be detected, and had biological activities such as anti-inflammatory, ion channel-blocking, immune-stimulation, antifungal, and antitumor properties [23]. Tupistroside is a group of bioactive molecules with pharmacological activity, and there was an intensive effort to the isolation of these valuable bioactive compounds in recent years [38]. The results obtained in this study showed that extracts of TPR can be considered a rich source of these molecules. At the same time, the results revealed that Asparagoside A can be isolated from TPR. This compound belongs to a class of phytochemicals with potential pharmaceutical effects [39]. Asparagoside A, extracted from Asparagus officinalis, has shown significant cytotoxic activity in human tumor cells [40]. So far, there are no studies available for the bioactive properties of Timosaponin A1. However, the analogue compounds of Timosaponin A1, including Timosaponin B-II and A-III, were analyzed for their biological functions. The previous studies showed that Timosaponin B-II had anti-inflammatory activity and Timosaponin A-III had anti-cancer activity [41], supporting that Timosaponin A1 could probably have bioactivity, but needs more investigation. Therefore, the results of this study show that the use of thermal processing can greatly shorten the time of traditional pickled radish and can also change the function of saponin (Figure 6).

## 3. Materials and Methods

### 3.1. Plant Material and Chemical Reagents

The radish, Raphanus sativus (550 ± 30 g), used in the experiment was purchased from Tainan Agricultural Products Marketing Co., Ltd., (Tainan, Taiwan) and has a complete production and sales history. The commercial pickled radish (CPR) was purchased from Taichung Jian Guo Market (Taichung, Taiwan). The production time of CPR, which was 7 days, 3650 days, and 7300 days, was named CPR A, CPR B, and CPR C, respectively. Methanol (Honeywell, Korea), vanillin (Katayama Chemical Co., Ltd., Osaka, Japan), 98% sulphuric acid (Katayama Chemical Co., Ltd., Osaka, Japan), and oleanolic acid (Sigma, Burlington, MA, USA) were purchased for the detection of saponin content. Formic acid (Daejung, Korea) and acetonitrile (Fan yuan Biotech Co., Ltd., Taichung City, Taiwan) were purchased for high-performance liquid chromatography. All solvents/chemicals were analytical grades and purity was more than 95%.

### 3.2. Sample Processing and Preparation of Extracts

The thermal processing radish (TPR) was processed at three different heating temperatures (70 °C, 80 °C, and 90 °C) with 90% relative humidity by a programmable constant temperature and humidity testing machine (CH-TH-5BP-A, E. Chung Machinery Company, Taoyuan, Taiwan), and the extraction procedure for TPR was based on the previous study and performed with some modifications [17]. All groups were analyzed for saponin content every 3 days during the processing period of 30 days. Then, the samples were lyophilized and ground to powder form for further studies. Dried TPR (1 ± 0.01 g) was soaked with 20 mL of 80% methanol (1:20, *w*/*v*) and maintained at 70 °C for 3 h in a water bath (R-2000S, Panchum Scientific Corp, Kaohsiung, Taiwan). The extracted solution was filtered and stored at 4 °C for further analysis. Moreover, CPR samples were purchased from Xiluo Agricultural Products Market Co., Ltd., compnay (Xiluo town, Yunlin, Taiwan) to be used as a reference to compare the final saponin content of samples.

### 3.3. Evaluation of Saponin Content and Kinetics of the Formation of Saponins

The saponin content of the radish extracts solution was determined using the method used by the previous study with slight modification [17,42]. Briefly, 0.1 mL of the extract was mixed with 0.1 mL of 8% (*w*/*v*) vanillin solution, and 1.0 mL of 72% H_2_SO_4_ solution was added. The mixture was incubated at 60 °C for 15 min and rapidly cooled to room temperature using an ice water bath. Finally, the absorbance was measured in a spectrophotometer (Thermo-1510, Thermo Scientific, Waltham, MA, USA) at 560 nm and compared to an oleanolic acids equivalents (OAE) calibration curve. The results are presented as mg OAE/g of dry weight (d.w.).

To calculate the order reaction, general kinetic models of zero-, first-, and second-order reactions were used to obtain the order of bioactivity reaction through Equations (1)–(3):

C − C0 = k × t(1)

ln (C/C0) = k × t(2)

(1/C) − (1/C0) = k × t(3)

where C is the compound content at a specific time/day, C0 is the compound content at time/day 0, k is the rate constant, and t is the storage time [43].

### 3.4. Analytical Mass Spectrometry

Liquid chromatography (LC) separations were optimized and eventually performed on a liquid chromatography–mass spectrometry (LC-MS) (Shimadzu LC-MS-8045 triple quadrupole mass spectrometer, Shimadzu Corporation, Kyoto, Japan) system using a reverse-phase analytical column (150 × 4.6 mm, 3 µm; C-18, Agela, Tianjin, China). The mobile phase used was water with 0.1% acetic acid (A) and acetonitrile with 0.1% acetic acid (B). An aliquot of 10 µL sample solution was injected into the high-performance liquid chromatography (HPLC) system, and the linear eluting gradient was as follows: 0–5 min at 10% B, 5–20 min at 10–50% B, 20–55 min at 50–100% B, 55–60 min at 100% B, and 60–65 min at 100–10% B at a flow rate of 0.4 mL/min, and the column temperature was maintained at 30 °C. For the electro-spray ionization–mass/mass (ESI-MS/MS) experiment, the positive mode MS conditions were set as follows: source voltage, 3.0 kV; cone voltage, 40.0 V; desolvation temperature, 350 °C; capillary temperature, 250 °C; nebulizing gas flow rate, 6.0 L/min; sheath gas (N_2_) pressure, 40 arb; Aux gas (N_2_) pressure, 10 arb; collision energy, 10 V; collision energy grad, 0.035 V/m. Mass spectra data were obtained with the full-scan mode for m/z in the range from 100 to 1000. Method validation was performed similarly to the protocol described in a previous study [36]. All data acquisition and analysis were performed using the AB SCIEX PeakView™ software 2.2 [44]. The saponin mass spectrometry database can be accessed at http://cpu-smsd.com (accessed on 21 October 2022) and identified the saponins in complex matrices [45].

### 3.5. Statistical Analysis

Each experiment was performed in a triplicate and completely randomized design (CRD). All values were expressed as means ± standard deviations and each sample was analyzed in triplicate (*n* = 3). Statistical data analysis was performed using one-way analysis of variance and implemented using dispersion analysis with SPSS 20 software (2011; IBM Institute, Inc., Chicago, IL, USA). Significant differences were determined using Duncan’s multiple range test at *p* < 0.05.

## 4. Conclusions

The results of this study show that the use of thermal treatment at 70 and 80 °C can effectively replace the traditional process of increasing the saponin of pickled radish. Additionally, the use of thermal treatment at 70 and 80 °C accelerates the structural transformation of saponin by increasing the reaction rate. This structural transformation can promote the formation of a more functional saponin structure from Tupistroside G to Asparagoside A and Timosaponin A1. Moreover, the kinetic analysis indicated that the thermal treatment above 80 °C may cause a significant decrease in the saponin content, and 70 °C can effectively promote the increase of the saponin content. The saponin content of samples treated at 70 °C reached the highest on day 12 and then decreased. Therefore, this study showed that appropriate thermal treatment conditions, in terms of time and temperature, can yield more saponin content with different functional components. These findings can provide a process reference for the agri-food processing industry to replace pickling to improve the functional properties of radish samples and can also expand the health application value of radish-based products by thermal treatment.

## Figures and Tables

**Table 1:** The saponin content of thermal processing radish (TP) and commercial pickled radish (CPR).

Sample Number	Processing Temperature (°C)	Time (Days)	Saponins Content (mg g^−1^)
CPR A	25	7	6.50 ± 1.46 ^e^
CPR B	25	3650	23.11 ± 1.22 ^c^
CPR C	25	7300	18.37 ± 1.11 ^d^
TPR	70	30	24.24 ± 1.01 ^bc^
	80	30	23.33 ± 2.32 ^c^

Results are expressed as means ± standard deviation (*n* = 3). Different superscripts in the same column indicate significant differences at *p* < 0.05. CPR: commercial pickled radish (commercial sample); TPR: thermal processing radish.

**Table 2:** Saponins value of k and R^2^ for zero-, first-, and second-order reactions during thermal processing of radish.

		Zero-Order Equation		First-Order Equation		Second-Order Equation	
	Treatment	k	R^2^	k	R^2^	k	R^2^
Saponins content	70 °C	0.1820	0.7181	0.0050	0.7507	−0.0001	0.7821
	80 °C	–0.5044	0.7126	−0.0188	0.6787	0.0007	0.6351
	90 °C	–0.8579	0.1054	−0.0387	0.4314	0.0019	0.7930

## Data Availability

Not applicable.

## Sample Availability

Samples of the compounds are available from the authors.
